# A Patient-Centered Mobile Health System That Supports Asthma Self-Management (breathe): Design, Development, and Utilization

**DOI:** 10.2196/10956

**Published:** 2019-01-28

**Authors:** Plinio Pelegrini Morita, Melanie S Yeung, Madonna Ferrone, Ann K Taite, Carole Madeley, Andrea Stevens Lavigne, Teresa To, M Diane Lougheed, Samir Gupta, Andrew G Day, Joseph A Cafazzo, Christopher Licskai

**Affiliations:** 1 School of Public Health and Health Systems Faculty of Applied Health Sciences University of Waterloo Waterloo, ON Canada; 2 Institute of Health Policy, Management, and Evaluation University of Toronto Toronto, ON Canada; 3 Centre for Global eHealth Innovation Techna Institute University Health Network Toronto, ON Canada; 4 Hotel Dieu Grace Healthcare Windsor, ON Canada; 5 Kingston General Health Research Institute Kingston, ON Canada; 6 Asthma Research Unit Kingston Health Sciences Center Kingston General Hospital Research Institute Kingston, ON Canada; 7 Ontario Lung Association Toronto, ON Canada; 8 Child Health Evaluative Sciences The Hospital for Sick Children Toronto, ON Canada; 9 Dalla Lana School of Public Health University of Toronto Toronto, ON Canada; 10 Institute for Clinical Evaluative Sciences Toronto, ON Canada; 11 Division of Respirology Department of Medicine University of Toronto Toronto, ON Canada; 12 Li Ka Shing Knowledge Institute St. Michael’s Hospital Toronto, ON Canada; 13 Institute of Biomaterials and Biomedical Engineering University of Toronto Toronto, ON Canada; 14 Western University London, ON Canada; 15 London Health Sciences Victoria Hospital London, ON Canada

**Keywords:** smartphone, asthma, self report, self-management, patient compliance, telemedicine, risk reduction behavior, internet, monitoring, physiologic, mobile applications

## Abstract

**Background:**

Uncontrolled asthma poses substantial negative personal and health system impacts. Web-based technologies, including smartphones, are novel means to enable evidence-based care and improve patient outcomes.

**Objective:**

The aim of this study was to design, develop, and assess the utilization of an asthma collaborative self-management (CSM) platform (*breathe*) using content based on international evidence-based clinical guidelines.

**Methods:**

We designed and developed *breathe* as a Web-based mobile health (mHealth) platform accessible on smartphones, tablets, or desktop with user-centered design methods and International Organization for Standardization–certified quality development processes. Moreover, *breathe* was envisioned as a multifunctional, CSM mHealth platform, with content based on international clinical practice guidelines and compliant with national privacy and security specifications. The system enabled CSM (patient, provider, and *breathe*) and self-monitoring of asthma patients through (1) assessment of asthma control, (2) real-time access to a dynamic asthma action plan, (3) access to real-time environmental conditions, and (4) risk-reduction messaging. The data collection protocol collected user data for 12 months, with clinic visits at baseline and 6 and 12 months. Utilization outcomes included user interactions with the platform, user impressions, self-reported medication use, asthma symptom profile, reported peak flow measurement, and the delivery and impact of email reminders.

**Results:**

We enrolled 138 patients with a mean age of 45.3 years to receive the *breathe* intervention. Majority were female (100/138, 72.5%), had a smartphone (92/138, 66.7%), and had a mean Asthma Control Test score of 18.3 (SD 4.9). A majority reported that *breathe* helped in the management of their asthma. Moreover, *breathe* scored 71.1 (SD 18.9) on the System Usability Scale. Overall, 123 patients had complete usage analytics datasets. The platform sent 7.96 reminder emails per patient per week (pppw), patients accessed *breathe* 3.08 times, journaled symptoms 2.56 times, reported medication usage 0.30 times, and reported peak flow measurements 0.92 times pppw. Furthermore, *breathe* calculated patients’ action plan zone of control 2.72 times pppw, with patients being in the green (well-controlled) zone in 47.71% (8300/17,396) of the total calculations. Usage analysis showed that 67.5% (83/123) of the participants used the app at week 4 and only 57.7% (71/123) by week 45. Physician visits, email reminders, and aged 50 years and above were associated with higher utilization.

**Conclusions:**

Individuals with asthma reported good usability and high satisfaction levels, reacted to *breathe* notifications, and had confidence in the platform’s assessment of asthma control. Strong utilization was seen at the intervention’s initiation, followed by a rapid reduction in use. Patient reminders, physician visits, and being aged 50 years and above were associated with higher utilization.

**Trial Registration:**

ClinicalTrials.gov NCT01964469; https://clinicaltrials.gov/ct2/show/NCT01964469

## Introduction

### Background

Asthma is a common chronic disease that poses a serious global health problem. In Canada alone, asthma affects 10.8% of Canadians [1]. Globally, the Global Burden of Diseases, Injuries, and Risk Factors Study estimated that 339 million people suffer from asthma, where asthma is the most common chronic disease among children [2,3]. However, 50% of patients with asthma are uncontrolled, leading to substantial personal and health system impacts [4-8]. In Canada, there are 150,000 emergency room visits and 60,000 hospitalizations triggered annually by asthma [9].

Collaborative self-management (CSM) is defined as “a system of coordinated healthcare interventions and communications for populations with conditions in which self-care efforts are significant” [10]. National and international guidelines and systematic review evidence recommend CSM, including a written asthma action plan, patient education, and regular clinical review [11-14]. CSM has been shown to substantially improve important patient and health system challenges, by reducing hospitalizations, emergency room visits, unscheduled visits to a doctor, absenteeism, nocturnal asthma symptoms, and significantly improving quality of life [14]. Moreover, a majority of patients prefer an active or collaborative role in their asthma management, particularly in the context of an asthma exacerbation [15,16]. Despite this strong evidence, these patient preferences, and consistent recommendations in international guidelines [11-13], CSM continues to be available to only a minority of patients (2%-11%) [5,17]. For these reasons, asthma is a chronic disease well suited for an examination of the transformative promise of smartphone mobile health (mHealth) apps in support of CSM.

Smartphones have become ubiquitous, and mHealth apps have the potential to transform elements of chronic disease management [18,19]. mHealth apps offer new opportunities for access to care, disease specific education, monitoring and disease management, personalized goal setting, adherence reminders, and communication. Requisite to the success of smartphone apps as new tools in the management of chronic diseases are a commitment to, and evidence of, user-centered design (UCD); development; and evaluation to ensure privacy, efficacy, and safety. Beyond the requirements of good design and development, the central question of whether patient-facing asthma apps that support CSM are efficacious remains unanswered.

### Objectives

We sought to design and develop a multifunctional, CSM mHealth platform for patients with Asthma, based on clinical content from international evidence-based guidelines, following a UCD process and then evaluate its utilization to inform iterative product improvement.

## Methods

### Overview

The *breathe* development program was structured in 2 main phases: (1) the design and development process for building the *breathe* mHealth platform including architecture, design, platform content, functional elements, user experience, and utilization (University Health Network REB 12-0102-AE and 12-0102-AE_Amendment) and (2) an evaluation of the patient outcomes by randomized controlled trial (RCT; Western University HSREB 102842, Queens University HSREB 6007261, ClinicalTrials.gov NCT01964469) and by a population-based cohort study. The utilization data reported in this manuscript are derived from the intervention (*breathe*) arm of the RCT. The RCT comparing conventional best practice plus the *breathe* platform with conventional best practice has been completed, and the main results are published in abstract form [20]. The focus of this manuscript is to share the design and development of the *breathe* platform, *breathe* utilization, and the user experience. The results of the RCT will be published in an upcoming manuscript.

#### Development Specifications of *breathe*

Specifications were developed collaboratively with Canada Health Infoway and included (1) a user-centered Web-based asthma self-management platform available on any Web-enabled device including mobile phone browsers and standard Web browsers on laptop, desktop, and tablet to ensure equitable access of the app; (2) patient access to their personal health information and electronic health (eHealth) records through connectivity with TELUS health space, which was a localized version of Microsoft HealthVault (Web-based personal health record developed by Microsoft); (3) alignment with national and provincial clinical and eHealth priorities, as per the Canadian Thoracic Society (CTS), Ontario Lung Association (OLA), eHealth Ontario (a provincial agency tasked with the implementation of Ontario’s public Electronic Health Record System), and the Ontario Ministry of Health and Long-Term Care; and (4) scalability to the provincial level and ability to be leveraged by other jurisdictions within Canada. Evidence-based best practices from the CTS Asthma guidelines [11] and the Global Initiative for Asthma guidelines [12] guided clinical content development.

#### The Development Team

*breathe* was developed by the Centre for Global eHealth Innovation at the University Health Network in collaboration with clinicians; researchers; and scientists from Western University, Queen’s University, Hospital for Sick Children, and the University of Toronto. The Centre is certified under International Organization for Standardization 13485, an international quality management system, to ensure the safety and quality of innovations. The mHealth platform development was guided by a 16-member interdisciplinary steering committee including asthma expert respirologists, certified asthma educators, population health scientists, knowledge translation experts, and eHealth experts. These experts were informed by 4 working groups: benefits evaluation, technical, consumer engagement (patients with asthma), and clinical. Working groups comprised a few members of the steering committee, along with additional individuals who contributed specific expertise such as consumers (patients with asthma), information technology professionals, and clinicians.

#### The Design Process

**Figure 1:**
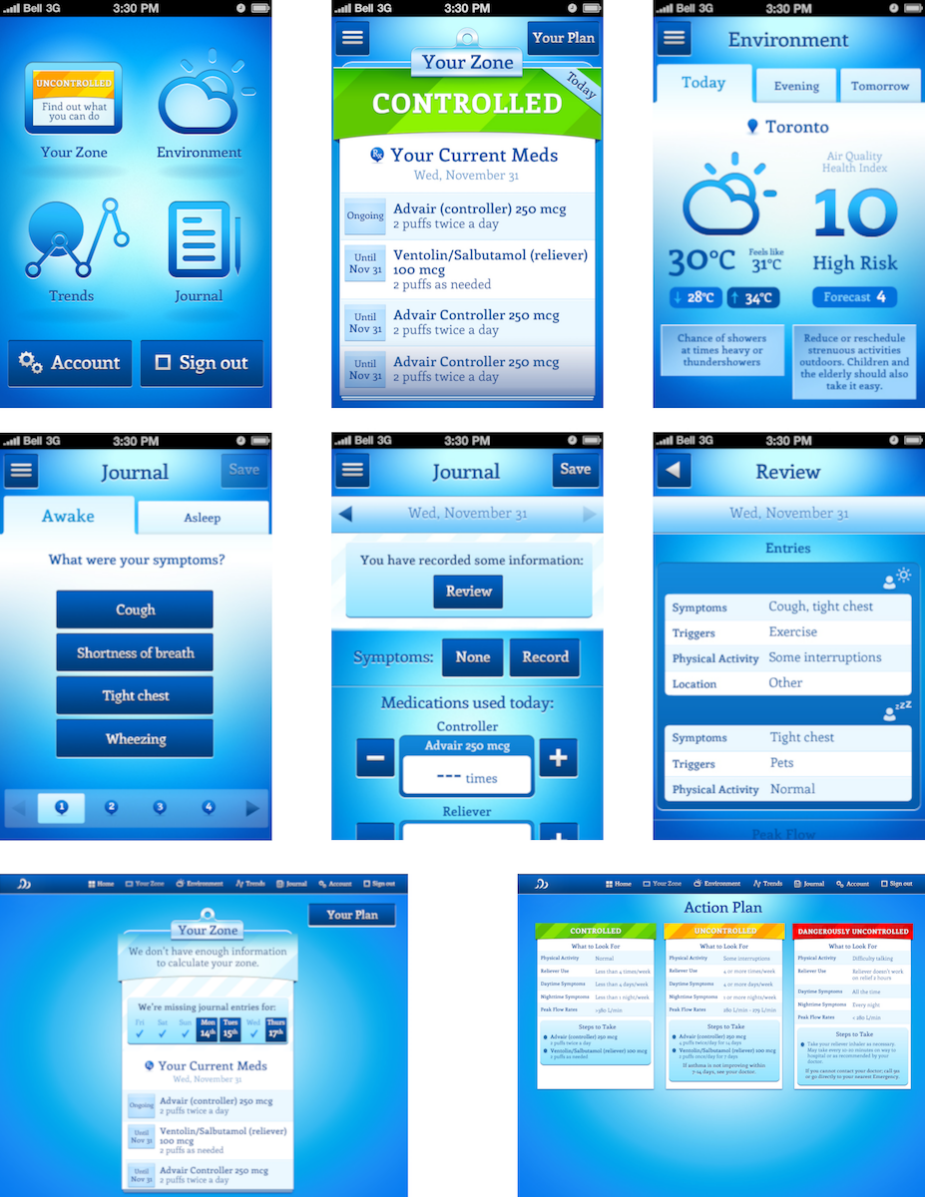
Examples of the various features designed for breathe. The first row provides examples of the main home screen, the current zone of control that the patient is in, and environment information. The second row provides examples of the journaling feature where users can report symptoms, medication intake, and review entries. The last row has examples of the desktop version of breathe, where the zone of control review and action plans are displayed. These are not actual plans, medications, or patient data but instead, prototypes of the breathe interface.

*breathe* (Figure 1) was designed using UCD methods [18,21,22], ensuring that the input and requirements of final users of the technology (patients, caregivers, and physicians) were included in the design process. The iterative UCD process included 11 interviews and 5 usability testing and cognitive walk-through cycles [21,22]. The semistructured interviews were conducted with representative end users (adults who have asthma) to test assumptions related to the use of a monitoring system as an intervention to enhance healthy self-management behaviors and disease-related decision making. These interviews employed a qualitative, ethnographic approach. Information was gathered and organized by extracting common themes identified by the participants. This initial user research provided the necessary evidence for the conceptualization and initial prototypes of the intervention, which was subsequently used in usability testing and walk-throughs. This UCD process explored the intuitiveness of the app and identified user preferences and expectations. Multiple cycles of cognitive walk-throughs and usability testing allowed the *breathe* team to improve the design based on user feedback and observed issues, focusing on the needs of the platform’s final users and avoiding the paradoxes of expertise [21]. The final design of the platform ensured that functionality was aligned with clinical needs and patient preferences and limitations.

### Evaluation of the Patient Experience, Platform Usability, and Utilization

#### Patient Recruitment

The utilization data reported in this manuscript are derived from the intervention (*breathe*) arm of the RCT designed to evaluate patient outcomes [20]. Participants were recruited from 6 primary care and 2 specialty asthma clinics in Ontario, Canada. A convenience sample of patients was self-identified after viewing posters in the clinic or invited to participate by clinic staff. The participating clinics were geographically distributed—for example, North, East, Southwest, and Central Ontario—with a range of urban and rural communities. All participants randomized to *breathe* had a baseline onboarding clinic visit where they were provided with *breathe* accounts, received a brief orientation, and completed a 6- and 12-month follow-up visit.

#### Platform Usability, Consumer Satisfaction, and Confidence

Overall, 2 customized consumer satisfaction questionnaires and the standardized System Usability Scale (SUS) [23] were administered at 6 months and 12 months post enrollment.

#### Measuring Platform Utilization

*breathe* was designed to collect usage data (in-app analytics) to enable data-driven design and evaluation. Information flowing through the *breathe* data server was logged and used as a part of this evaluation. The *breathe* server tracked medications prescribed to patients, self-reported medication use, actual peak flow compared with personal best or normal, action plan zone of control, general access, and email notifications sent by the system. Each entry to the database was identified with a unique user ID and time stamped to enable further analysis.

#### Statistical Methods

The statistics reported in this manuscript are primarily descriptive. We reported counts and percentages for categorical variables as well as means and SDs for continuous variables or pseudocontinuous variables derived as means of multiple ordinal questionnaire items. We used the Wilcoxon rank-sum Test to compare the number of weeks with at least one login during the 52 weeks between groups defined by age, college education, smartphone use, and baseline Asthma Control Test (ACT) score. Age groups were defined as less than 50 years versus aged 50 years and above because it approximately divided the population in half.

## Results

### mHealth Platform Architecture of *breathe*

**Figure 2:**
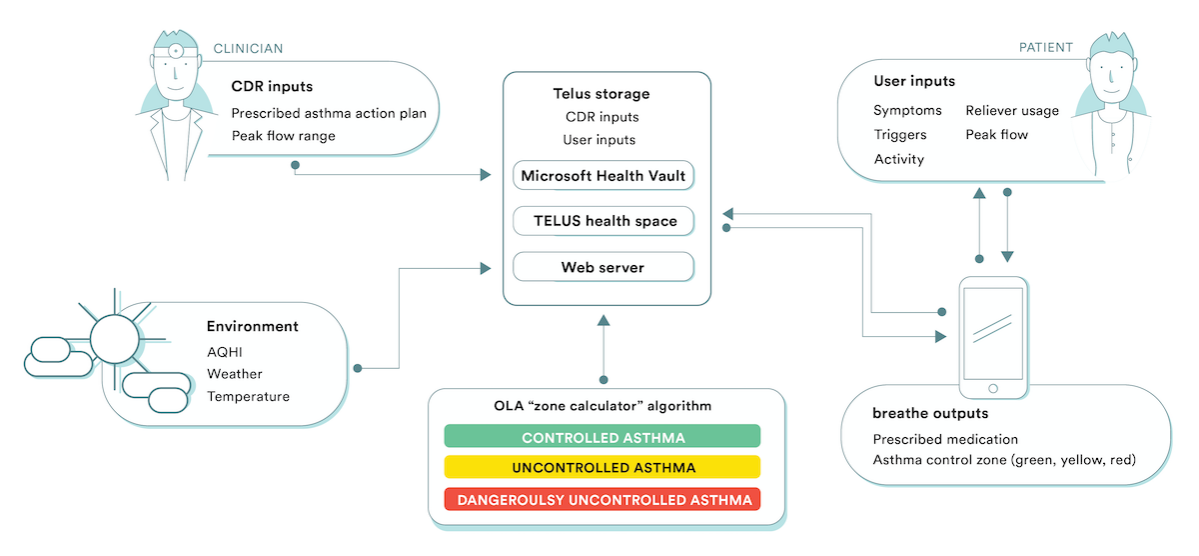
Architecture of the *breathe* platform (Ontario Lung Association [OLA], Air Quality Health Index [AQHI], Clinical Data Repository [CDR]). In cases where peak flow was part of the action plan, peak flow ranges were entered by the provider. Patients were responsible for entering peak flow measurements.

*breathe* is a Web-based mHealth platform that utilizes HTML5 and responsive design allowing a single version of the platform to be accessible on any device (smartphone, tablet, or personal computer; see Figure 2). Moreover, *breathe* interfaces with TELUS health space, where it receives up-to-date medication and peak flow ranges from the integration of clinical data repositories (electronic medical records). Furthermore, *breathe* retrieves real-time environmental conditions directly from Environment Canada, which include current and forecasted weather conditions, in addition to the Air Quality Health Index (AQHI) with relevant risk-reduction health messaging from Health Canada. The AQHI is a simple 1 to 10 scale designed to help individuals understand air quality, the impact of poor air quality on their health, and what actions to take to minimize health risks [24].

### Functionality of *breathe*

The health care provider developed an *asthma app prescription* in a collaborative triad of patient, provider, and app. The health care provider determined the patient’s asthma medications, their individualized action plan by zone, and peak flow ranges for control zone calculations (if applicable). The *breathe* platform did not advise on the selection of medications and did not create the action plan. This remained a physician responsibility. Integrating with TELUS health space offered patients the option to share *breathe* data with family members and other health care providers, which could be accomplished through the health space Web-based profile. The *breathe* features can be seen in Figure 1. Each of these features was designed to engage users and collect relevant data to support self-management, as described below.

#### Journal

The Journal feature allows patients to track daily symptoms, record reliever and controller medication usage, and log peak flow measurements. The historical review feature allows users to look back at previous journal entries and peak flow values entered.

#### Your Zone

The journal entries feed an integrated asthma control algorithm at the *breathe* server, based on the CTS Asthma Guidelines [11,12] that analyzes patient inputs and immediately advises the patient of their current zone of control: (1) green zone—in control, (2) yellow zone—uncontrolled, or (3) red zone—dangerously uncontrolled. The zone of control assessment is paired with the actionable recommendations from patients’ personalized asthma action plan. The zone of control is dynamic, immediately updated with any new journal entries and resets after the action plan has been executed, ensuring a tailored and customized intervention to the patient [25]. Patients were notified of changes on their zone of control through the app dashboard and in the Your Zone section.

#### Trends

Data visualization and analysis of several trends, including identified triggers, control zone, and peak flow values, were available to users. An example of the usefulness of this feature is that trigger frequency reported back to patients may enable patient insights into which triggers to avoid in the future.

#### Environment

This feature provides real-time current and forecast of location-specific (based on users’ input about their location) environmental conditions including temperature, humidity index, weather forecast, and the AQHI with specific poor air quality risk-reduction health messaging.

#### Account

This feature includes a variety of options including changing default (7:00 pm EST) time and email address to receive emailed medication adherence reminders and setting a location for location-specific environmental information. Email adherence reminders were automatically generated based on predefined rule-based logic including a welcome email, *check-in* emails for users not accessing the platform within 7 days, and daily adherence reminders for controller medications. There was no limit to the daily reminder emails; however, we designed the system to try to mitigate fatigue by creating approximately 30 different body messages that were randomly emailed to the user. A demonstration of how the *breathe* platform works can be found in the Canada Health Infoway website, with a detailed description of functionalities and platform capabilities.

### Patient Population

A total of 344 patients were recruited into the RCT between October 31, 2012, and March 31, 2014, of whom 171 were allocated to the *breathe* intervention arm. Consent was withdrawn (n=10) or we were unable to find the patient to consent for data transfer (n=23) in 33 patients, leaving 138 patient that could be used in this analysis. Complete platform utilization data were available in 89.1% (123/138) participants, and 12-month usability and satisfaction questionnaires were available for 86.2% (119/138) participants. The majority of the 138 patients were women 72.5% (100/138), mean age 45.3 (SD 15.8) years, and 97.1% (134/138) were Caucasian. Of these participants, 66.7% (92/138) had a smartphone, and the majority 83% (76/92) reported being *comfortable* or *very comfortable* using it. Patients recruited had a mean ACT score of 18.3 (SD 4.9), suggesting well- to somewhat-well-controlled baseline asthma [26].

### *breathe* Usability, Patient Satisfaction, and Confidence (12-Month Data)

Usability was evaluated by the SUS, a validated composite measure, which is scored from 0 to 100, with higher scores representing greater usability (Table 1). The *breathe* system scored 71.1 (SD 19.9) at 12 months indicating good usability, as defined by Bangor et al [27]. The mean of 7 ease-of-use questions scaled from 1-very difficult to 5-very easy was 4.1 (SD 0.9). A majority found *breathe* components useful and were satisfied with the design. (Table 1)

Satisfaction was evaluated using 5-point Likert scale responses, 1-strongly disagree, 3-do not know or neutral, 5-strongly agree. A total of 63.8% (74/116) of patients agreed or strongly agreed that the *breathe* app was helpful in the management of their asthma. Moreover, 65.2% (75/115) of patients were confident that the *breathe* app was correct when it presented the patient’s asthma action plan zone of control. Furthermore, 49.6% (58/117) of participants agreed or strongly agreed that they would continue to use the app after the study if it remained available (Table 1).

### Actual *breathe* Usage

The 123 patients in the intervention arm with utilization data accessed *breathe* 19,678 times (3.08 times per patient per week, pppw), reported symptoms in their diary 16,357 times (2.56 times pppw), reported medication use 1922 times (combined use of reliever and controller; 0.30 times pppw), and reported peak flow measurements 5864 times (0.92 times pppw). Total counts can include patients accessing the platform multiple times in the same day.

**Table 1:** Usability questionnaire.

Usability and user satisfaction of *breathe*			Statistics at 12 months
**Satisfaction**			
	**The** ***breathe*** **app that was provided to me by the clinic is helpful in the management of my asthma, n (%)**		
		Disagree or strongly disagree	21 (18.1)
		Agree or strongly agree	74 (63.8)
	**I would continue to use the** ***breathe*** **app if it were available to me after the study, n (%)**		
		Disagree or strongly disagree	30 (25.6)
		Agree or strongly agree	58 (49.6)
	**I was confident that when the** ***breathe*** **app was correct when it assessed my asthma zone of control, n (%)**		
		Disagree or strongly disagree	16 (13.9)
		Agree or strongly agree	75 (65.2)
**System Usability Scale (score range 0-100), mean (SD)**			71.1 (19.9)
	**Evaluation of specific functional components of** ***breathe*** **(on a scale of 1-very difficult, 3-don't know or neutral, and 5-very easy)**		
		Ease of use: mean of 7 questions (n=119)	4.1 (0.9)
		Usefulness: mean of 12 questions (n=118)	3.6 (0.9)
		Design of components: mean of 12 questions reported (n=119)	4.2 (0.7)

**Figure 3:**
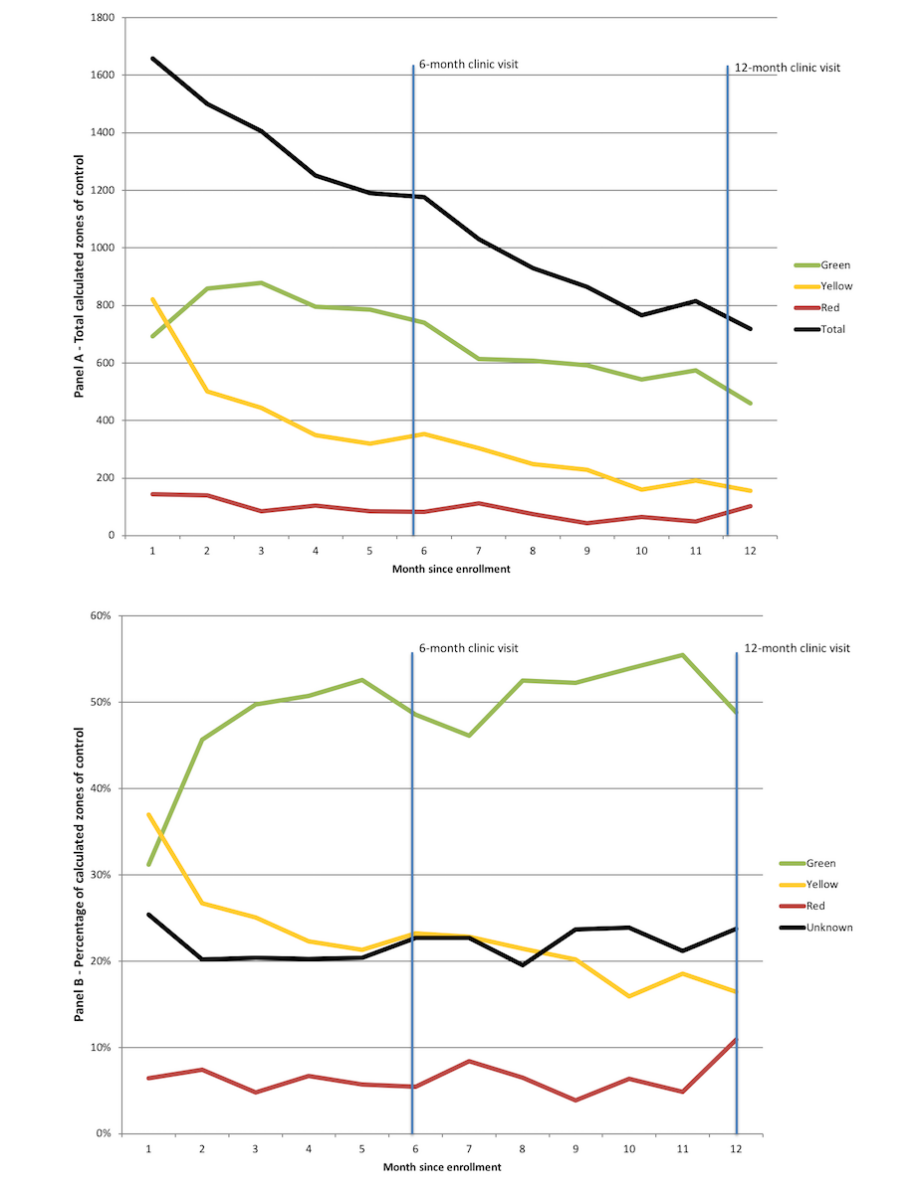
Panel A: Total calculations of zone of control calculations per month of the intervention calculated from enrollment Panel B: Percentage of zone of control calculations per month of the intervention.

*breathe* calculated patients’ action plan zone of control 17,396 times (2.72 times pppw). Patients were most often in the green zone of control (47.71% of calculations, 8300/17,396), followed by yellow zone (23.90%, 4158/17,396) and red zone (6.40%, 1110/17,396). In 22.00% (3826/17,396) of the calculations, *breathe* did not have enough information to return a zone of control back to the patient based on the programmed algorithm in the *breathe* platform (Figure 3).

*breathe* sent 50,939 emails (7.96 times pppw) to remind participants to take their controller medications or to return to the platform after 7 days of no usage. *breathe* did not log email responses potentially generated by the users.

**Figure 4:**
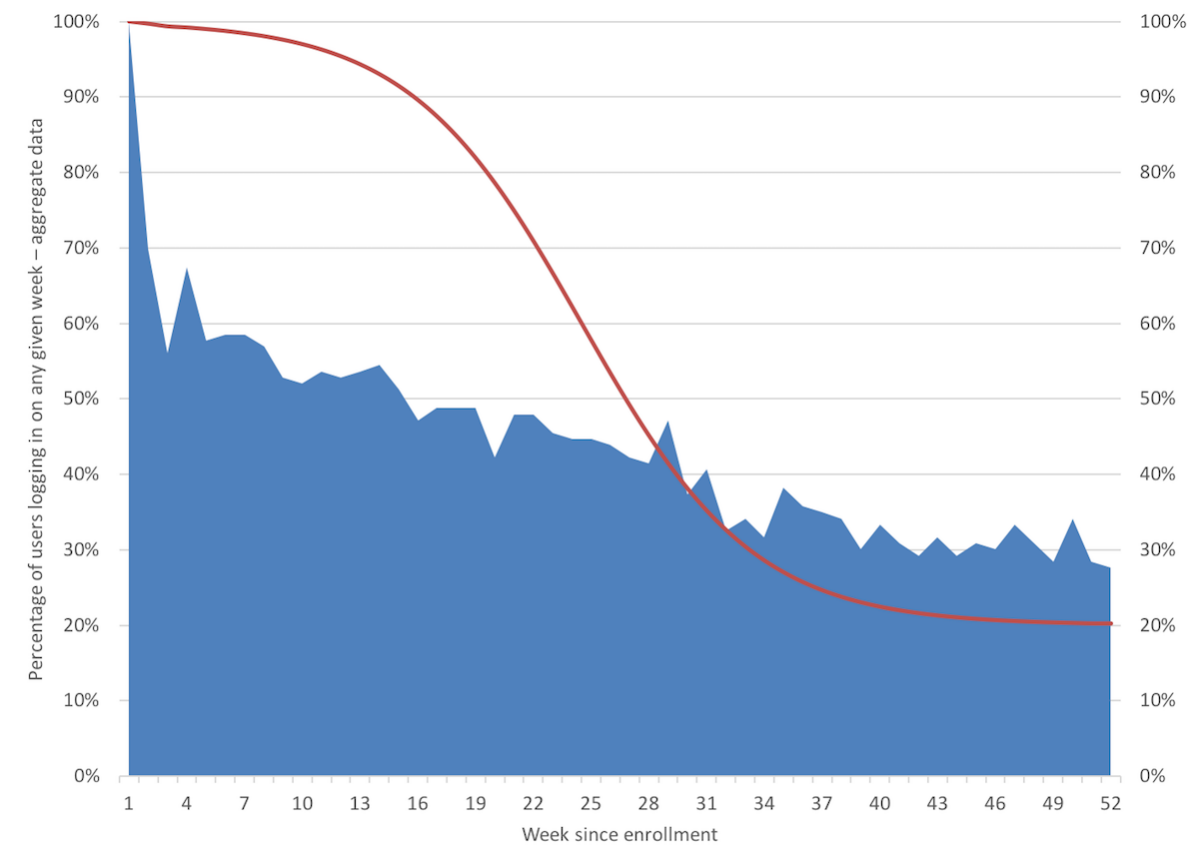
Attrition in breathe use throughout the 12-months of the study, with Eysenbach attrition curve plotted as a reference.

Tracking patient log-ins to the platform demonstrated a fall in use within the first 4 weeks of initiation and thereafter a standard decay in usage (Figure 4), whereby 67.5% (83/123) of the participants used the platform weekly initially and only 57.7% (71/123) used the platform in week 45. Figure 4 presents our patient log-in data along with Eysenbach attrition curve [28].

Further utilization analysis demonstrated patterns of use that related to patient behavior, *breathe* functionality, or the interaction of both.

**Figure 5:**
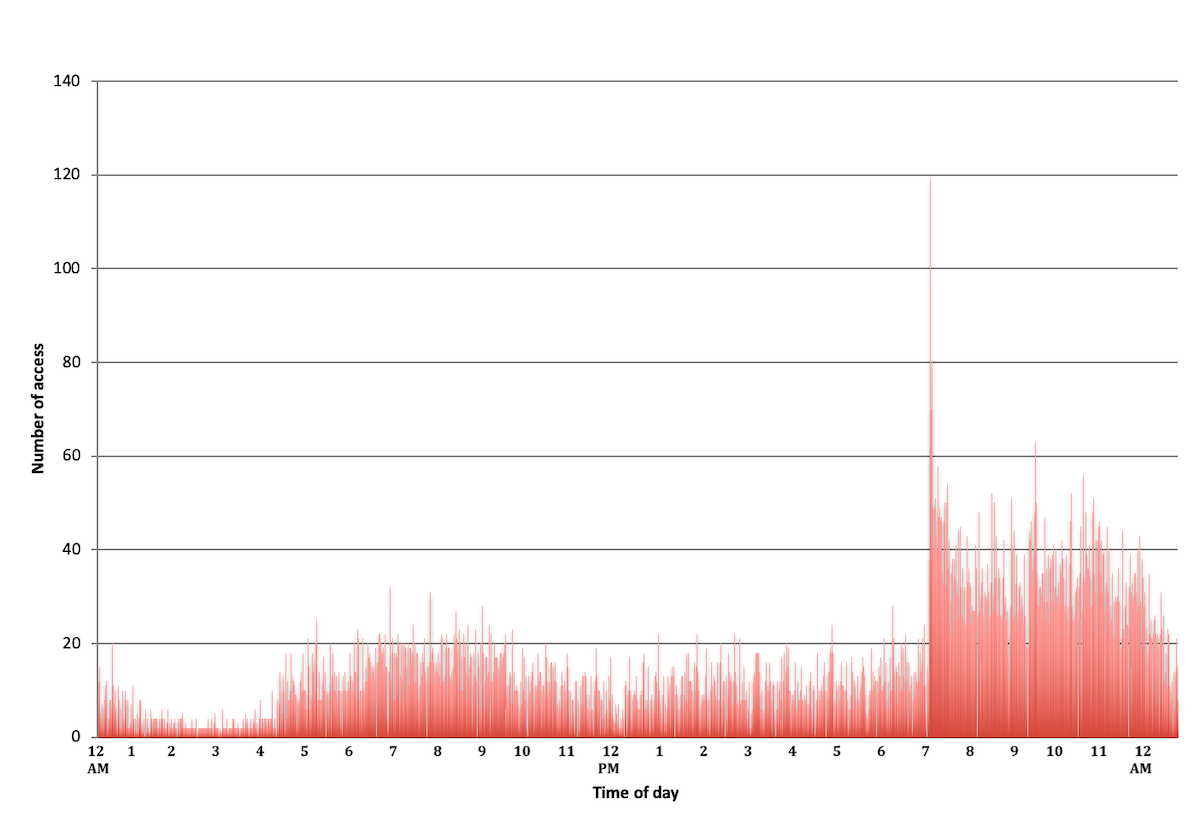
App use tracked by number of logins by time of day exploring the effectiveness of reminders. Note that automatic app reminders are default to send around 7:00 pm.

**Figure 6:**
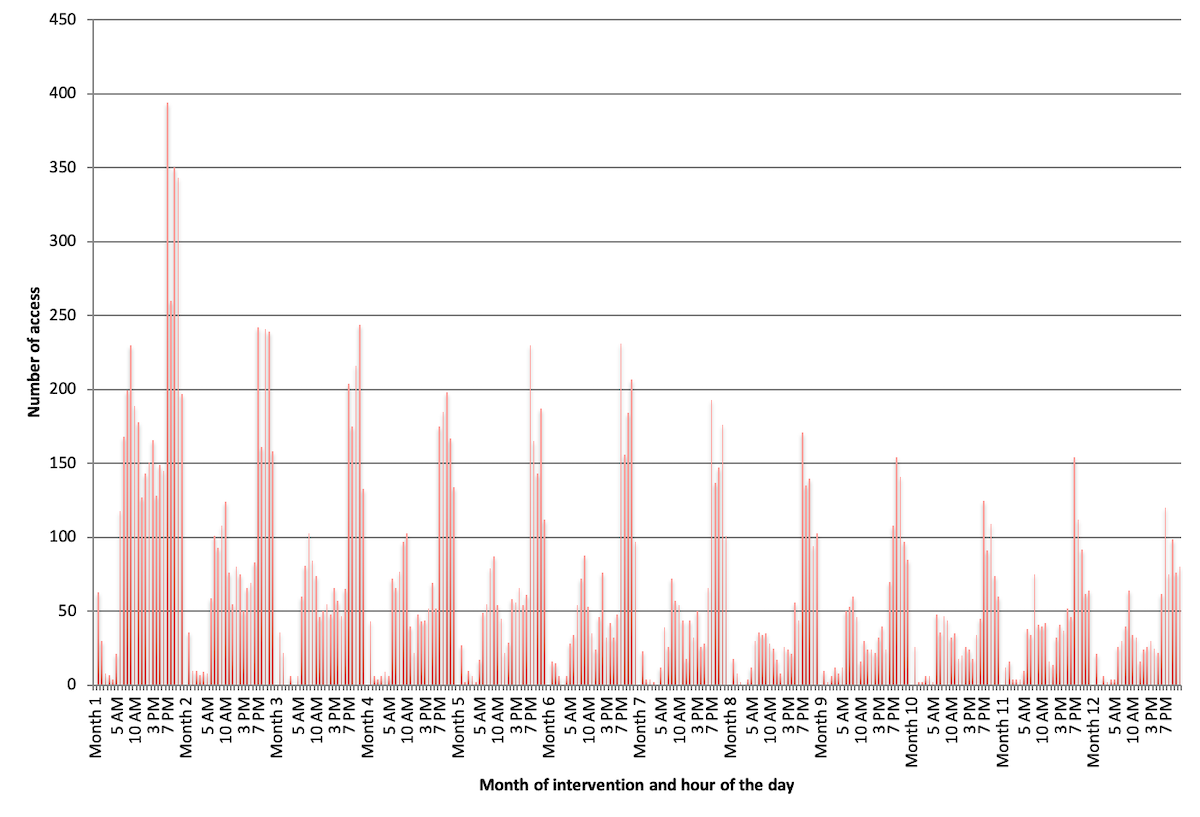
Sustained effect of email reminders on app use over the 12 months of intervention.

**Figure 7:**
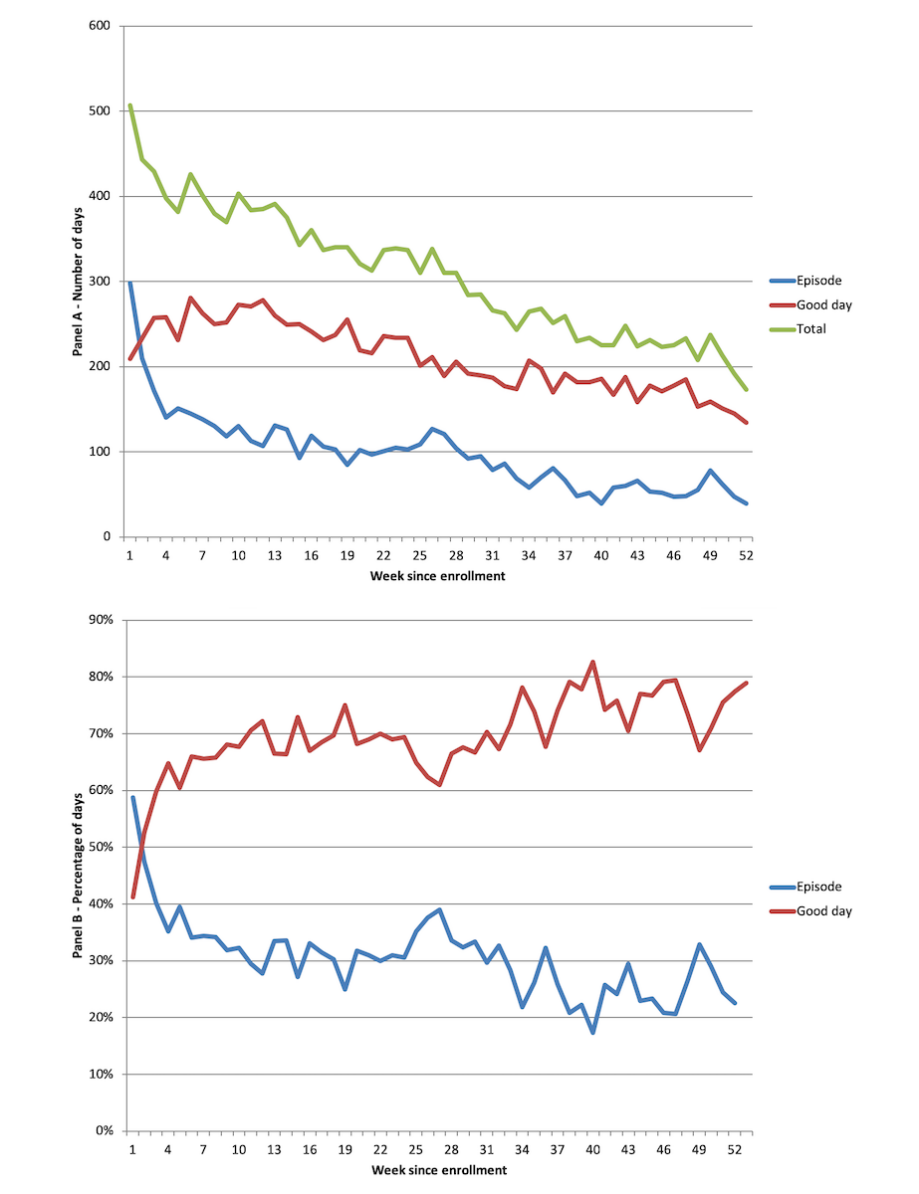
Panel A: Number of reported “good days” (no symptoms) and symptom episodes since enrollment. Panel B: Percentage of reported “good days” (no symptoms) and symptom episodes since enrollment.

**Figure 8:**
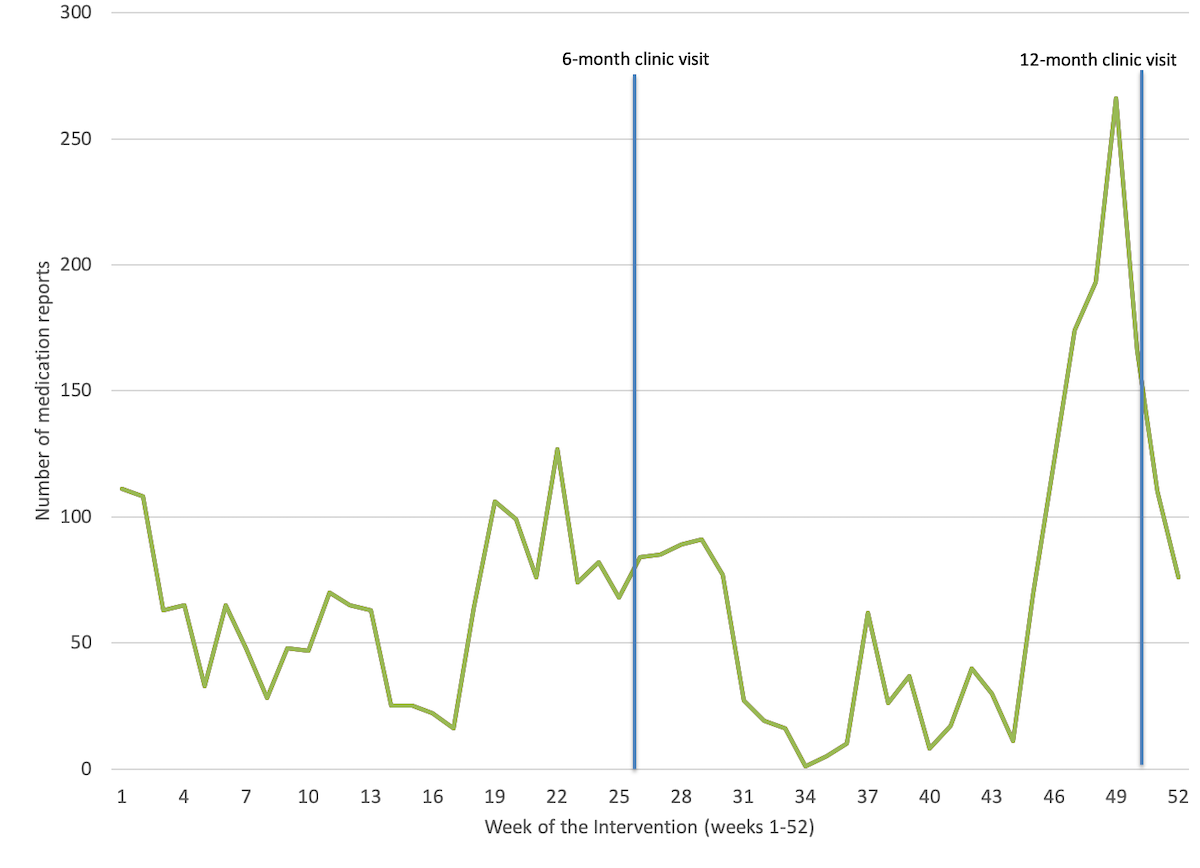
Self-reported controller medication use showing the effect of clinic visits (surveillance effect) on self-reporting behavior (clinic visits were scheduled at 6 and 12 months from the beginning of the intervention).

- *Time of day:* Analyzing log-ins by time of day revealed 2 periods of increased utilization (Figure 5). First, there was higher platform use between 5:00 am and 10:00 am, which corresponds to the time of the day when most patients are waking up and preparing for their day. Second, there was a dramatic spike in utilization just after 7:00 pm, the default time of day when the *breathe* system email reminders were automatically sent by the app server. This finding was sustained each month over the 12 months of the study (Figure 6).
- *Symptom reporting:* Evaluation of the *Journal* functional element within the platform revealed approximately twice as many reports of good days (a day without symptoms) compared with days with symptoms (Figure 7), which aligns with our expectations for well-controlled asthma.
- *Scheduled physician visits:* Finally, based on controller medication recording, there was an increase in platform utilization in weeks 26 and 52, corresponding to scheduled follow-up visits (Figure 8).

The post hoc analysis of patient factors that may have influenced utilization including age, education level, smartphone use, and asthma control is presented in Table 2. Only age (≥50 years) was associated with higher utilization.

**Table 2:** Utilization by patient characteristics, indicating the number of weeks with at least one log-in during a 52-week period.

Group		Weeks (n)		Mean (SD)		*P* value^a^	
**Age (years)**							
	<50		73		18.2 (17.9)		<.001
	≥50		49		30.1 (18.2)		—^b^
**College educated**							
	No		34		21.6 (20.8)		.42
	Yes		88		23.5 (18.2)		—
**Use smartphone**							
	No		40		23.8 (19.7)		.66
	Yes		82		22.6 (18.6)		—
**Baseline Asthma Control Test score**							
	<20		65		22.7 (18.7)		.97
	≥20		57		23.2 (19.2)		—

^a^*P* value from Wilcoxon rank-sum test.

^b^Not applicable.

## Discussion

Despite a decade of mHealth app development, there remains a limited body of evidence demonstrating improved health outcomes with apps [29-31]. Currently, there are 3 published RCTs evaluating patient-facing, multifunctional asthma apps developed to support CSM. Liu et al [32] showed increased quality of life, increased use of controller medications, improved lung function (8%), and decreased emergency service use. Merchant et al [33] demonstrated the effectiveness of the Propeller Health Asthma Platform at reducing the use of short-acting β-agonist (SABA) by 0.41 activations per day (vs 0.31 control), increasing the number of SABA-free days by 21% (vs 17% control). Conversely, Ryan et al [34] found that mobile phone–based monitoring did not improve asthma control or patient self-efficacy compared with a paper-based monitoring system. Our experience with an asthma app prototype in a recent pilot study revealed a high level of satisfaction with the app (80% of users viewed the app positively, with the majority wishing to continue using the app after the study), regular participation in self-management, and improvements in asthma-related quality of life [35]. The central question of whether mHealth platforms that support asthma management such as *breathe* are efficacious remains unanswered. We assert that good platform design is a precondition to posing and answering this question.

### Usability, Satisfaction, and Confidence

Patients had a high level of satisfaction with the individual design components of *breathe*. They rated *breathe* usability as good on the validated SUS and high on standard Likert scales. A central function of the *breathe* platform was to present patients with a real-time dynamic action plan based on their symptoms and peak flow entries. The app effectively returned a dynamic zone of control calculation back to patients. *breathe* patients were confident that the calculated assessment was accurate. By objectively measuring control, *breathe* resolves a long-standing barrier to action plan utilization in the community, the barrier of inaccurate control assessment by patients. Patients who overestimate control will not activate their action plan as prescribed and thus not experience the substantial associated clinical benefits [5-8].

The *breathe* mHealth platform was an important facilitator of teachable moments and acted as an unidirectional communication bridge between providers and patients in the community through the delivery of 50,939 reminder email messages and communicating asthma control and care recommendations through the platform 19,678 times. An examination of utilization suggests that patients responded to these notifications by accessing the app after the reception of these emails, and patient questionnaires indicate that they had confidence in the care and control recommendations.

### App Usage

The goal of UCD is to create and sustain a certain level of adherence to the platform, as adherence is a prerequisite to positive behavioral change and improved health outcomes. Despite good ratings for ease of use and a high degree of satisfaction with the *breathe* system, actual platform use declined substantially over time, which in general aligns with reviews describing attrition rates in eHealth deployments [28,36-38]. In his seminal viewpoint paper “The Law of Attrition,” Eysenbach argues the need for a *science of attrition* and recommends that usage metrics be measured, analyzed, and discussed to identify reasons for attrition [28]. The *breathe* utilization curve differs substantially from Eysenbach specifically related to a dramatic fall in utilization in the first 4 to 6 weeks. We evaluated factors associated with increased or decreased platform utilization.

We considered that decreased utilization (attrition) in this study might have been related to population and design characteristics, including technology savviness, patients with relatively good disease control, infrequent physician monitoring, or because patients achieved their expected outcomes (or the correct *digital dose* of the intervention).

#### Technology Savviness

All participants had access to either a smartphone or a computer. Although, 55.2% (76/138) of our population had a smartphone and reported being comfortable or very comfortable with its use, one-third did not have a smartphone and therefore accessed the platform by laptop, desktop, or tablet. We considered that the nonsmartphone subset may have been less technology savvy, contributing to the decline in utilization and particularly may have contributed to the sharp decline in the first 4 weeks. However, our post hoc analysis did not find an association between utilization and having a smartphone.

#### Age and Education Level

We considered that younger age and higher education level might have an impact on utilization. We did not find an association between utilization and education level. In a post hoc analysis, we were able to demonstrate that being aged 50 years and above was significantly associated with higher utilization. Although general app use is normally greater in a younger population, we speculate that our participants over the age of 50 with a chronic disease may have had a higher level of concern about their chronic disease and potentially find more value in health-related apps than a younger population. We observed that increased utilization was associated with time of day, anticipated physician visits, and email reminders.

#### Good Disease Control

Patients in this study had relatively well controlled asthma as indicated by high baseline ACTs and a high percentage of good days when compared with episode days. We did not have a specific engagement strategy to motivate patients to return to the platform when they were feeling well. Failure to engage the users in moments of disease stability has been described by other authors as a critical factor affecting attrition across diseases [39-41]. However, our post hoc analysis did identify an association between utilization and high versus low scores on the ACT.

#### Physician Monitoring

In this study, patients were evaluated by a physician only twice after enrollment. Infrequent monitoring may have increased the attrition rate. Increased *breathe* platform utilization was associated with upcoming 6- and 12-month clinic appointments. An increase in eHealth utilization in response to anticipated clinical review has been described by Mohr et al as supportive accountability [42] and by others [43,44] as a strong factor influencing sustained adherence. The surveillance effect has a direct influence on how engaged patients are with the platform and how much they adhere to the intervention. Along the same lines, eHealth platforms that provide some level of feedback and peer support appear to demonstrate better adherence rates [45]. The need for regular clinical review to motivate platform adherence aligns with the literature supporting written asthma action plans, where efficacy requires regular clinical review [14]. The finding related to increased medication reporting at 6 and 12 months also suggests that for most of the year, medication use was underreported. Self-reported medication use may underreport actual use [46]. New Bluetooth-enabled smart inhalers that automatically log medication use [47] will be considered in the future development of *breathe*.

#### Patients Achieved Their Expected Outcomes

Patients were satisfied with *breathe*, and 63.8% (74/116) agreed or strongly agreed that “the *breathe* application is helpful in the management of my asthma.” Thus, it is possible that after an interval, having achieved their personal goals, patients no longer felt a need to use the platform.

#### Email Reminders

Increased *breathe* platform utilization was associated in time with email adherence reminders. Others have identified reminders as powerful design features to increase adherence and engagement with eHealth platforms [48], to alert participants of important events [19,49], or to alert them of aspects of the treatment they have missed [45]. Although alarm fatigue has been described in long-term interventions, wherein reminders lose their impact over time [50,51], we demonstrated a sustained effect of reminders over the 12 months.

#### Usage Analysis Summary

Patterns of usage analysis identified physician visits and email reminders as strongly associated with utilization. A post hoc analysis identified being aged 50 years and above as significantly associated with higher utilization.

### Limitations

The population studied was a convenience sample from primary and specialty clinics with a dedicated asthma program, and at the time of enrollment, patients had relatively good asthma control. As such, patients’ evaluation of the app and their utilization patterns may not be representative of the general asthma population. Since this project was completed, native apps have largely supplanted Web browser–based apps such as *breathe*. The improved performance of native app platforms may positively impact utilization and reduce attrition.

### Conclusions

We followed UCD methods to develop *breathe*, a multifunctional asthma CSM platform with content based on international clinical practice guidelines, compliant with national privacy and security specifications, to support patients as active participants in chronic disease management at home, work, and in the community. *breathe* enabled self-management and self-monitoring of asthma patients through assessment of asthma control, real-time access to a dynamic action plan, environmental conditions display, and air quality risk-reduction messaging. Individuals with asthma reported good usability and high satisfaction levels and had confidence in the platform’s assessment of asthma control. We embedded in-platform analytics, evaluated utilization, and examined the utilization patterns in the context of known patient characteristics. We related increased utilization to physician monitoring, email reminders, and age 50 years and above. Looking to the future, embedded app analytics combined with data-driven design will enable real-time evaluation of mHealth platforms, enabling innovators to execute design improvements during the deployment of the technology.

### Lessons Learned or Future Considerations

As we iterate development of the *breathe* platform based on lessons learned, we will seek to (1) leverage the surveillance effect of in-platform or in-person patient-physician contact to support utilization, (2) create a specific strategy to engage patients when they are feeling well and to reengage as they become unwell, (3) create a strategy to support adherence specifically for asthma patients aged less than 50 years, (4) integrate automated logging technology (smart or connected inhalers) to capture actual medication utilization, (5) leverage the sustained impact of patient reminders on utilization, (6) create a more interactive experience to enhance platform use, (7) utilize embedded app analytics that provide continuous evaluation of usage to enable the execution of design improvements during platform deployment, and (8) develop the next version of the *breathe* platform with a native iOS or Android app.

## Abbreviations

ACT: Asthma Control Test
AQHI: Air Quality Health Index
CIHR: Canadian Institute for Health Research
CSM: collaborative self-management
CTS: Canadian Thoracic Society
eHealth: electronic health
mHealth: mobile health
OLA: Ontario Lung Association
pppw: per patient per week
RCT: randomized controlled trial
SABA: short-acting β-agonist
SUS: System Usability Scale
UCD: user-centered design
